# Codified Hashtags for Weather Warning on Twitter: an Italian Case Study

**DOI:** 10.1371/currents.dis.967e71514ecb92402eca3bdc9b789529

**Published:** 2016-07-05

**Authors:** Valentina Grasso, Alfonso Crisci

**Affiliations:** National Research Council of Italy, Consorzio LaMMA - CNR Ibimet, Sesto Fiorentino, Florence, Italy; National Research Council of Italy, Istitute of Biometeorology, Florence, Firenze, Italy

## Abstract

Introduction: During emergencies increasing numbers of messages are shared through social media platforms becoming a primary source of information for lay people and emergency managers. For Twitter codified hashtagging is emerging as a practical way to coordinate messages during emergencies and quickly identify relevant information. This paper considers a case study on the use of codified hashtags concerning weather warning in Italy in three different regions.

Methods: From November 3rd to December 2nd 2014, tweets identified by the 3 codified hashtags #allertameteoTOS, #allertameteoLIG and #allertameteoPIE were retrieved, collecting a total of 35,558 tweets published by 7361 unique tweets authors, with the aim to assess if codified hashtags could represent an effective way to align formal and informal sources of information during weather related emergencies. An auxiliary R-package was built to lead the analytics used in this study. Authors performed a manual coding of users, hashtags and content of messages of all Twitter data considered.

Results: Content analysis showed that tweets were overwhelmingly related to situational updates, with a high percentage containing geo-location information. Communication patterns of different user types were discussed for the three contexts. In accordance with previous studies, individuals showed an active participation primarily functioning as information hub during the emergency.

Discussion: In the proposed cases codified hashtags have proven to be an effective tool to convey useful information on Twitter by formal and informal sources. Where institutions supported the use of the predefined hashtag in communication activities, like in Tuscany, messages were very focused, with more than 90% of tweets being situational updates. In this perspective, use of codified hashtags may potentially improve the performance of systems for automatic information retrieval and processing during disasters.

Keywords: social media, emergency management, Twitter, severe weather

## Introduction

**1.1 How social media reshaped emergency communication**

Social media are widely used for personal and public purposes as a way to enhance communication and foster relations 1. Social media platforms like Facebook, Twitter and Instagram have enormously increased the amount of information exchanged every day on the internet, especially during particular events. Emergencies makes no exception, making it possible for the general public to play an active role during a disaster by contributing with information exchange. The crowdsourcing of relevant information through social media is considered important for emergency communication. “Accepting the public as a legitimate an equal partner” 2 is recognized as a best practice in crisis communication and nowadays social media makes it possible for the community to be part of the crisis communication response 3. Many researches have demonstrated the key role played by Twitter during emergencies in the last years; examples are the 2007-2008 California Wildfires 4, Haiti earthquake 5 ^,^ 6 , Australian floods 7, Hurricane Ike 8, Hurricane Sandy 9, Red River floods 12 ^,^ 43, Colorado floods 10, Thailand floods 11. Research 12 shows that information shared on Twitter may improve situational awareness and help people on the ground to collect important information.

During a disaster people with a mobile phone can potentially send information from the field improving situation monitoring; if mobile networks are functioning properly (sometimes they collapse in catastrophic events or are saturated by too many simultaneous users) people may redistribute information acting as a hub for communication diffusion. Through social networking sites, like Twitter or Facebook, the news of a crisis or an accident can be shared by millions of people at a diffusion rate unknown ten years ago. In some cases, words and messages from lay people, people-like-you, are trusted even more than those produced by the official media 13. Approaches like those of crisis informatics 14 ^,^ 15 consider how Information and Communication Technologies (ICT) can improve crisis response by integrating official responders and members of the public. During a disaster people act as information seekers, using information from family and peers as a mean to integrate and sometimes validate those issued by official sources 16.

**1.2 Twitter, hashtags and emergencies**

The amount of information exchanged online can be overwhelming and we may not be able to separate what is relevant from the noise. In Twitter the use of hashtags tends to reduce this effect. Hashtags are words prefixed with the # symbol that works as message label helping to associate Twitter posts to specific events or discussions. The first use of a hashtag emerged as a tagging convention by Twitter users during an emergency, the California wildfires in 2007. Nonetheless, only a small percentage of Twitter messages contain one or more hashtags 17.

Hashtags are very useful to categorize messages and more organizations are now managing hashtags in a proactive way, identifying specific words or acronyms to label events like a conference, meeting, sports match or TV shows. The use of hashtags may help with the dissemination of information during emergencies but in many cases there is a lack of coordination between government agencies and volunteers in hashtag adoption so information diffusion is not helped 18. During emergencies a consistent use of hashtags would help users to quickly access information about the event without randomly searching Twitter for relevant messages. Hashtags have also been recognized as a positive factor for retweeting behaviour 19. For a hashtag to go viral its adoption by authority and influencers, like media accounts is very important 20.

In recent years codification of Twitter hashtags emerged as an issue in the field of emergency management. The Philippine government was one of the first public agencies to set up a strategy on codified hashtags in 2012 to be used during emergencies 21. The government declared they had “promoted the use of unified hashtags (#rescuePH and #reliefPH) to monitor, track, and consolidate information before, during, and after a natural disaster strikes”. As reported by Meier^21^ , the Philippine government suggested a clear convention to create new hashtags by using the local name of the storm in combination with the country acronym PH (e.g. #YolandaPH). An official statement was also distributed to the media and the public to adopt unified hashtags when tweeting about weather-related reports. The Government declared that unified hashtags were very useful in central urban areas where Twitter is more widely used but also in more decentralized ones where Twitter messages conveyed by means of unified hashtags were useful for enforcing communication between government, media and NGOs. That codified hashtags are an important issue in disaster response is confirmed by the publication Hashtag Standards For Emergencies issued in 2015 by the UN Office for the Coordination of Humanitarian Affairs, UNOCHA, where standardization of social media hashtags is recognized as a policy that could have major impact on integrating big-crisis data into emergency response. Like the Philippine government strategy, the publication suggests the adoption of a codified syntax to generate new hashtags for next future disasters 22 .

**1.3 Italy: codified hashtags for weather warning**

The Genoa flash floods on November 4^th^ 2011 was one of the firsts severe weather events in Italy where social media started to play a role in emergency management. Six people died during the flooding that submerged the largest city on Italy's north western coast and hundreds of buildings and properties were damaged. For the first time in Italy Facebook and Twitter were massively used to organize volunteers and help the city to cope with the disaster. Following the example of Crisis Commons^44^ meetings in which people discuss the role of technology in humanitarian assistance and disaster relief, in the aftermath of the Genoa flooding (2012/2013) two Crisis Camps were organized in Bologna, Italy, with the aim of opening a discussion with emergency management professionals, volunteer, researcher, public administration communicators and journalist on how to effectively use social media during emergencies. One of the main outputs of the meetings was a proposed “syntax” for codified hashtags to be used in emergencies. In November 2013, the hashtag #allertameteoSAR was proposed by a citizen, an influencer, to coordinate conversation and aids on Twitter during the floods that hit Sardinia. In January 2014 one of the meeting participants (@capitanachab) published the blog post “20 hashtags for a participated civil protection” 23 (http://capitanachab.tumblr.com/ post “20 hashtags per una protezione civile partecipata”) with a catalogue of twenty hashtags to be used during regional weather warning. These are a list of hashtags generated by combining “allertameteo” (weather warning) with the first three letters of the region. The reason for the regional connotation in the configuration of Italian weather warning systems is that regional meteorological monitoring centres are in charge of local warning for severe weather events. No government recommendations or documents were released to trigger the tactical hashtags adoption during emergencies; it was nothing more than a commitment by some local governments and active citizens to start codified hashtags diffusion. The first regions to actively use them turned out to be those where severe weather events occur most often and flood risk is high.

Some researches have already investigated the use of social media during an emergency in Italy; Parisi et al. (2014) 24 presented some case studies and in particular two weather related events: floods in Sardinia and snowfall in Bologna. The Sardinia case study showed a first analysis on the use of hashtags #allertameteoSAR to crowdsource information from the ground to manage emergency and post-emergency phases. A more local analysis was the one by Giglietto and Lovari (2013)^25^ on the Florence snowfall that showed an active role of Florence municipality Twitter account @comunefi in establishing the emergency hashtags. This paper is a first analysis of the adoption of the weather warning codified hashtags in three different regional contexts.

## Research and methodology

The work presents a comparative analysis of the three codified hashtags for weather warning during November 2014 where three Italian regions are involved: Liguria, Tuscany and Piedmont. The aim of the investigation is to assess if and how codified hashtags could represent an effective way to align informal and formal sources of information during weather related emergencies. Following this purpose, we analysed the messages that have been exchanged on Twitter micro-blogging platform during the selected period of time to identify the amount of information that those hashtags were able to attract, to characterize most active types of users, most used hashtags and classify the messages’ content to gain insights into the type of information exchanged. In particular content analysis was meant to assess the potential increase of on-topic tweets and of messages increasing situational awareness, compared to similar researches carried out in different contexts (Starbird et al. 2010^43^ ; Starbird and Palen 2010^45^ ; Vieweg al. 2010^12^; Hughes et al. 2014^8^; Sutton et al 2014^46^ ; Bonnan-White et al 2014^42^ ; Soriano et al. 2016^47^ ). Findings may be useful to improve information retrieval and processing during disasters, particularly those weather related 26 .

We compared three collections of tweets identified by codified hashtags during a period when several severe weather events occurred. The dataset considered is based on the codified hashtags #allertameteoPIE (integrated by #allertameteoPM) (N=2461), #allertameteoTOS (N =3165), #allertameteoLIG (N =29332). Tweets were collected during the period November 3rd to December 2nd 2015. Search resulted in 35,558 tweets and 7361 unique tweet authors.

**2.1 Meteorological background**

During November 2014 severe weather events (heavy rain and violent thunderstorms activity) occurred in north-western areas of Italy. As well documented on maps of the European Emergency Response Coordination Centre - EU ERCC web portal 27, in many meteorological national 28 and regional reports 29 ^,^ 30 ^,^ 31 ^,^ 32 ^,^ 33 and in a research paper 34, these events led to flooding different coastal cities, mainly in Liguria (Genoa), but also in Tuscany (Carrara) and Southern Piedmont. The dates of the three main events were: 3-6, 9-13 and 15-19 November 2014. The corresponding harvesting period of tweets also include another event, less severe and without significant impacts, at the end of November (27-11/ 01-12). During the period the Italian Civil Protection Department issued 9 red severe weather warnings in Liguria and 3 in Tuscany; 4 orange severe weather warning in Liguria, 5 in Tuscany and 6 in Piedmont (red is the maximum warning level).

The following image, provided by DPC Dewetra System (Italian Civil Protection Department) is a map of cumulated rainfall for November 2014 showing a clear view of weather pressure due to precipitation on the area of investigation.

**Map of cumulated rainfall for November 2014, Dewetra System, Italian Civil Protection Department:**
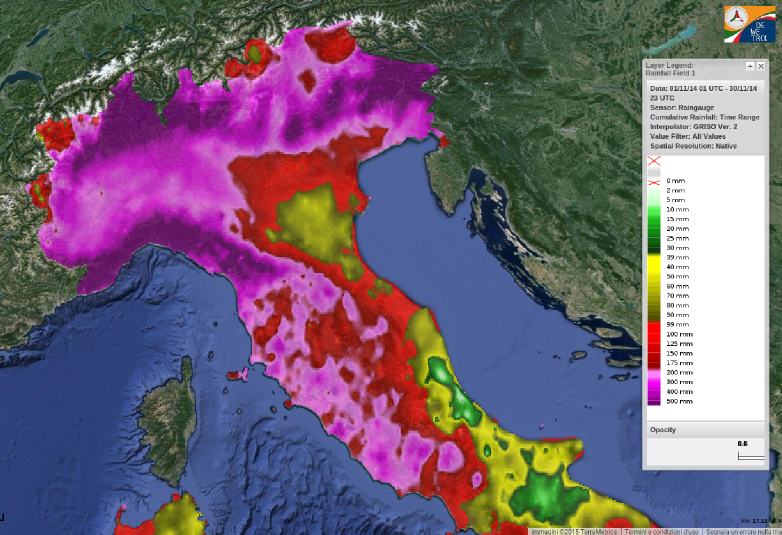

**2.2 Dataset and Methodology**

Tweets were collected by querying the Twitter Stream API that is the platform’ application which allows messages and users data harvesting as they are available in the client public timeline. The three regional codified hashtags for weather warning used for Twitter querying were: #allertameteoTOS for Tuscany, #allertameteoLIG for Liguria and #allertameteoPIE integrated by #allertameteoPM for Piedmont. A double querying parameter was used for #PIE because during the considered period both hashtags were proposed on Twitter as codified hashtags for Piedmont region.

To approach the analysis of these three datasets we started by defining each collection of tweets as a Twitter “channel” of information. A Twitter channel is defined as a time ordered collection of tweets obtained by the Twitter API by using a set of query parameters like hashtags, user accounts or simple word combinations; the time ordered tweets set obtained is named channel and for analogy could be interpreted as an informative content flow semantically linked to related events. A Twitter channel is an efficient way to monitor conversations and facilitate content data mining on defined topics or events identified by the structured combination of search parameters. Relevant metrics of the Twitter channels investigated were performed by using a dedicated R package developed for the work and released publicly on the web repository: https://github.com/alfcrisci/rTwChannel 35

Querying the Twitter API from November 3rd to December 2nd 2014 for the three codified hashtags a set of 35558 tweets was collected; three channels were created as shown in Table 1.

**Table 1: channels of the codified hashtags:** Channel identification and items

Channel identification	Querying parameters	Italian region	Number of collected tweets
#LIG	#allertameteoLIG	Liguria	29932
#TOS	#allertameteoTOS	Tuscany	3165
#PIE	#allertameteoPIE and #allertameteoPM	Piedmont	2461

Through the R-package each channel was analysed by using the common social-media metrics and also the daily distribution of tweets during the period considered. Channel’s metrics comparison was used to assess different levels of propagation of the codified hashtags within the regional contexts. To give a robust data analysis framework, authors also qualitatively examined and coded the main features of Twitter communication: users, hashtags and content of tweets. A coding scheme was customized to fit the investigation aims.

***Users annotation***

To better describe the channels and identify the communication pattern of different categories of users a manual annotation was made of recovered Twitter accounts. The aim was to classify users into main categories and accordingly verify their participation and active role in the codified hashtags. For classification of users we manually coded the whole set of unique authors by labelling accounts depending on their affiliation, as declared in the profiles’ description available on Twitter. We considered five classes of unique users deemed as relevant for weather related emergency management and fitting the purposes of this work: *Institution* (governments and public agencies); *Media* (tv, radio, news and online media); *Weather* (weather forecasting services or weather enthusiast associations); *Volunteers-NGO* (NGOs active in the field of rescue and emergency management); *Individuals* (accounts of not affiliated individuals; not belonging to any of the above).

By identifying classes of unique users with similar mission and role we compared their communication patterns on Twitter.

***Hashtags annotation***

We also performed a manual annotation of the set of hashtags extracted from each channel. Hashtags coding was intended to identify the popularity of semantically close hashtags used to derive information on which semantic domain users assign relevance to during emergencies. Identifying what kind of information users label as relevant is important for hashtags recommendation during weather warnings.

For each regional channel all hashtags were extracted and manually annotated as belonging to one or more of these classes: *Emergency*, expressions related to emergency warning, damage, severe weather impacts (like flood, mudslide; death reports); *Places,* geographic names like region, county, city, village, suburb, river or point of interest (such as the name of an airport or hospital); *Institution*, government, governors, municipality, mayor, public agencies; *Media*, words identifying media actors or products (TV-shows, TV-news, newspapers); *Weather*, words related to meteorology and forecasting; *Mobility,* words related to street name, motorway, railway and trains; *Social media*, words referring to the social media domain; *Time,* expression of temporal interval; *Others,* none of the above. Within the class *Places* a sub-class was also identified a sub class named *Outside_places* (different for all channels), to identify names of places located outside the regional domain of the dominant codified hashtag of the channel. Due to Twitter text limitation and users’ shorthand, locations were sometimes expressed in the tweets by means of acronyms, like FI for Florence or GE for Genoa; in this work hashtags expressed by acronyms were not annotated as belonging to the class Places.

Because Twitter hashtags cannot include space characters some hashtags turned out to be a combination of two or more words up to a real sentence. In this work composite hashtags were annotated twice, depending on the semantic domain of each lexical component. Because the hashtags annotation was intended mainly to gain a better knowledge about the semantic domain users assign relevance to during emergencies, in Twitter communication, hashtags composed of a combination of words reported a double annotation. For example a hashtag like #alluvionegenova obtained a double annotation, *Emergency* and *Places*.

As many studies recognize 7 ^,^ 36 ^,^ 37 ^,^ 38 a hashtag performs multiple functions: it serves as a bookmark of content and a symbol of community membership; in the case of adoption of codified hashtags the community function is supposed to be performed by this whereas the other hashtags included in tweets are supposed to mainly serve as a bookmark of content. As a result, to have a classification of hashtags in categories related to weather, emergency and geographic names it is important to evaluate which other relevant hashtags arose from the channel communities.

***Content annotation***

As last step we performed a content analysis of the native tweets of each dataset. Our aim was to identify and to measure the classes of information shared on Twitter by mean of the codified hashtags, in order to assess whether they may function as effective channels to convey formal and informal sources of information during emergency. Following similar works (43 ^,^ 46) for content coding were considered only messages from unique authors who posted more than 3 tweets over the considered period. Of the whole 7534 native tweets we coded a sample of 7039 tweets. Only this *Refined DataSet* (RDS) was considered for message’s content analysis.

We identified a set of eleven categories to describe the information communicated in each tweet, paying particular attention to those contributing at increasing situational awareness, considered as “*an individually, as well socially cognitive state of understanding the ‘big picture’ during a critical situation*” (Vieweg, 2010^12^). We discussed the coding scheme used in similar works and tested its fitting on the considered data; we also performed an independent analysis of the dataset looking for contents useful from the point of view of emergency management. Categories we considered for coding tweets were: *Advice* (how to cope with the emergency, safety precautions, local emergency numbers to call, advice on how to tweet; websites to follow); *Warning* (tweets about warning issued); *Hazard location/impact* (information on hazard localization or reporting about flood or weather impacts on specific location); *Weather* (information about weather conditions when directly described within the tweet); *Transport Conditions* (updates on road conditions; road closures; airport or public transport malfunctioning); *Evacuation and Closures* (message about closures/opening of public services, schools and scheduled events); *Damage/Injury reports* (reported damages on infrastructures or casualties); *Reassurance* (updates of action from first responders and volunteers’ activity on the ground); *Resources* (a shared resource, url, picture or video, related to weather or flood update; *Comments* (personal comments, questions, blames) and *News reports* (media resources shared by users). Works by Starboard et al. (2010)^43^ and Hughes (2014)^8^ guided the identification of categories concerning situational update; two more categories were added to classify media contribution and comments shared by the public broadening the general understanding of emergency impact on the population.

Some tweets were coded with more than one category and thus were considered as two messages in the analysis. The reliability of annotations was tested by using Cohen’s Kappa^48^ calculated comparing message categorisations performed by the two independent panels; an acceptable value was reached with a score of 0.9.

## Results and discussion

We began by examining general features of the channels’ dataset by looking at their composition and main features; we then looked at the daily Twitter activity for each channel; we analysed participation pattern by comparing the Twitter activity of different classes of annotated users. We examined the hashtags adopted in the three channels and the categories of information posted by the different Twitter users to make an assessment about codified hashtags adoption in these case studies.

The dataset is composed of 35,558 total tweets, main metrics are reported in Table 2.

**Table 2: main features of the three channels:** 

Channel	Total tweets	Retweets (RT)	Native tweets	Ratio tweets/RT	Unique users	Native tweets users	percentage of active users %	Ratio tweets/user
#LIG	29932	24017	5915	4	5782	1124	19%	5
#TOS	3165	2249	916	2.4	822	172	21%	4
#PIE	2461	1758	703	2.5	757	129	17%	3

The vast majority of tweets, 84%, are related to the channel #allertameteoLIG, #allertameteoTOS represents 9% of the whole set and #allertameteoPIE 7%. This is understandable due to the fact that there were several flash floods in Liguria during that period. On November 10th the town of Chiavari was flooded by some minor rivers. On November 15th the city of Genoa (with a population of 880,000 in city and suburbs) was flooded by the river Polcevera and different areas of the city were damaged; it was the second devastating flash flood in Genoa during Autumn 2014, so public attention was already very high.

To describe the channel we considered both the total number of tweets, retweets and replies collected and the subset composed of native tweets, defined as original tweets written by unique Twitter authors. In all three channels the majority of the dataset is made up of retweets, contributing for 70-80% of the total tweets. A high retweeting rate is recognized, in fact, as typical behaviour during a disaster event on social media 39 responding to people’s need to make information available. The #LIG channel also has the highest ratio between active users and total tweets, with a ratio of 5 (5 retweets per tweet) against 4 and 3 in #TOS and #PIE; the higher participation of #LIG is understandable due to the wider and heavier impacts of severe weather on several cities.

One of the metrics considered was the number of unique users participating in the #allertameteoXXX conversation: #LIG presents the highest number with 5782 unique users, compared to 822 in #TOS channel and 757 in #PIE. The majority of these users participated only by retweeting messages; only 20% of users were truly writing messages. This rate is quite similar for all three channels, slightly higher in Tuscany (21%).

**Table 3: mentions, hashtags, replies and URLs in tweets for the three channel:** 

Channel	Total tweets	Native tweets	Hashtags	Mentioned users	URLSs in tweets	Replies
#LIG	29932	5915	947	1077	2653	395
#TOS	3165	916	277	212	521	22
#PIE	2461	703	218	122	418	24

Replies were very rarely used, only 1% of all tweets.

Another feature considered was the amount of tweets containing web links (URL’s), as external contents or images uploaded on Twitter as links. Researches suggest that the presence of a URLs in a tweet is a sign of information richness and is recognised as a key element for situational awareness during a crisis^12^.

Almost 50% of native tweets in the 3 channels contained one or more URLs, ranging from 45% in LIG channel to 59% in #PIE channel. This high number is not surprising if we consider that these channels generated by codified hashtags are intended as a way to share information related to warning and emergencies published on the internet. The ratio Tweets/Retweet and the percentage of tweets with a URL is perfectly in line with “crisis event” features as suggested by the classification of Bruns (2012) 39 .

Tweets including mentions are less numerous than those with URLs: in #LIG and #TOS they reach 28% of native tweets, in #PIE only 13%. Citation habits are discussed in section 3.3.

The set of hashtags used in the stream is quite big. It is worth noticing that very few tweets contained only the specific channel codified hashtag: 63 for #LIG, 6 for #TOS and only 2 for #PIE; confirming that hashtagging behaviour is very user dependant

**3.1 Time distribution of channel activity**

Daily distribution of tweets give an idea of the channel activity, that is very related to severe weather events occurring in the period. Looking at the daily distribution of the dataset in Figure 2 it is quite clear that activity is registered when a warning is issued, peaks are when weather is severe and harmful, there is no activity when no warning is issued. Tweets collected for each channel correspond with the size and impact of the weather events. The different channels anyhow show some differences. While the #LIG channel reaches highest twitter activity in general its distribution is quite concentrated on event’s peak days with little activity in other periods; #PIE and #TOS channels show a more regular level of activity.

**Daily distribution of tweets and retweets for each channel and weather warnings issued:**
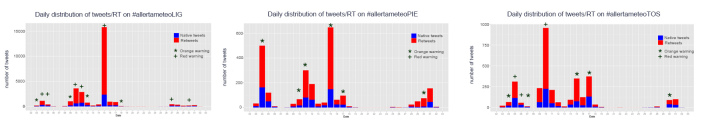
Blue bars visualize tweets, red ones retweets. "+" character indicate that a Red warning for severe weather was issued; "*" character that an orange warning was issued.

**3.2 Users participation by categories**

To better understand communication pattern of similar Twitter authors, unique users were classified in five categories (Institution, Media, Volunteers-NGO, Weather, Individuals), as earlier mentioned in 2.2. Table 4 shows active users for each category in the three channels and Figure 3 shows the contribution of each category to the dataset of every channel.

**Table 4: users activity rate by category in the three channels:** 

	#allertameteoLIG		#allertameteoTOS		#allertameteoPIE	
Users category	Tweet and retweet	Percentage of native tweets	Tweet and retweet	Percentage of native tweets	Tweet and retweet	Percentage of native tweets
Institutions	301	47%	703	58%	57	30%
Media	1401	15%	273	49%	468	45%
Volunteers - NGO	227	13%	82	30%	103	23%
Weather	1129	44%	536	19%	282	69%
Individuals	26874	19%	1571	16%	1551	17%

**Tweets contribution by users category:**
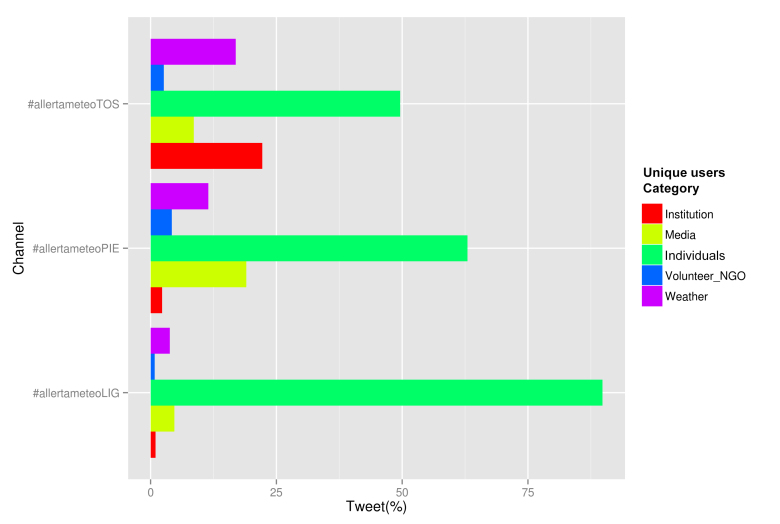
****Bars represent the percentage of tweets published by the corresponding category of unique users, in each channel.

Some differences are easily visible looking at the graph in Figure 3. In #LIG channel Individuals were the most active class contributing 90% of tweets; the category was also dominant in #TOS and #PIE. #TOS shows an important contribution of institutional users, the more active category after Individuals, followed by Weather. In #PIE and #LIG the most active class of Twitter accounts, after Individuals is Media, followed by Weather. In all channels participation of Volunteers is very limited. This could be explained by the fact that during emergencies volunteers and NGOs are active in the field and probably have no social media staff that regularly post messages.

To gain an enhanced view we also analysed the percentage of native tweets within each category (Table 4). This information is interesting to better define the behaviour pattern of different classes of users on Twitter and see the difference in the three contexts. In all channels we noticed that Individuals contribution was mainly expressed in retweeting, with only 16-19% of their messages being native tweets. This is in line with the literature reporting that citizens on social media act as information hub during emergencies 40. The Institution category, which played a very active role in #TOS channel, shows a different communication pattern in the three channels, with the highest native tweets rate in #TOS (58%) and lowest in #PIE (30%). During emergencies institutions are supposed to be the most important and trusted source of information; however a low level of activity in #LIG and #PIE cannot be interpreted that institutions were inactive on Twitter but only that they did not fully adopt the codified hashtag. In Tuscany the codified hashtag was more supported by the regional weather service, local civil protection offices and municipality, thus gathering more institutional accounts on #+TOS channel (in Tuscany the first use of #allertameteoTOS is dated January 31st 2014, by regional weather service Twitter account @flash_meteo). Media category made great use of retweets in #LIG, with respect to #TOS and #PIE, mainly due to the fact that they frequently retweeted citizens (see following section on citation behaviour). Weather category contributed with a high native tweets percentage in #PIE (69% of all tweets were native), in #TOS and #LIG their participation is expressed more in retweeting behaviour (81% of Weather tweets were retweets in #TOS and 56% in #LIG). Differences in communication pattern were also due to the more damaging floods impacting in Liguria cities like Genoa and Chiavari.

The balance between native tweets and retweets may be used to identify different user approaches 41: *annunciative* *approach*, posting mainly native tweets, or *disseminative* *approach*, posting mainly retweets. Institutions adopted an annunciative approach in Tuscany, while in Liguria and Piedmont the approach was more disseminative.

**3.3 Citation behaviour: mentioning and retweeting**

Another important metric we analysed to understand communication patterns in the three contexts was that of citation habits: retweets and mentions. As shown in Figures 4 and 5 we employed users coding to understand interactions between relevant categories. The network graphs present mentions and retweets amongst categories of users in the three channels.

**Mentions engagement by category of users:**
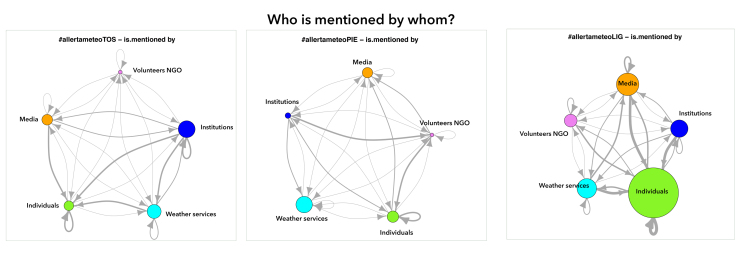
The circle dimension represents the number of received mentions; arrows direction indicates the relation “is mentioned by"; the thicker the line is, the higher the mentions received.

**Retweets by category of users:**
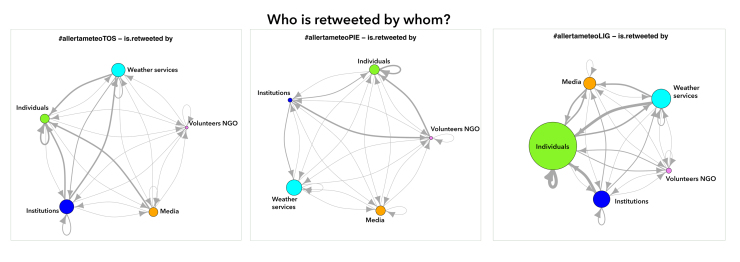
­The circle dimension represents the number of received retweets; arrows direction indicates the relation “is retweets by"; the thicker the line is, the higher the retweets received.

An important metric for understanding the channel is to establish which users are the most cited within the community created by the hashtag, measured by the number of @replies and retweets they receive. These are the accounts perceived by the hashtags community as more important and worthy of engaging with 41. Our analysis proposes this metric at category level, to better understand interactions between the classes of users.

Generally the most active category of users for retweets and mentions is Individuals, with some differences in the three channels. In #LIG Individuals mentioned and retweeted mainly accounts in the same class; this is also confirmed by the top three mentioned and retweeted authors, who in #LIG channel are individuals (see Table 1. in Appendix 1). In Liguria in fact members of the public supported the core of the conversation, their tweets amounting to 90% of the whole #LIG dataset. Individuals as citizens mentioned first other citizens, followed by Weather, Institution, Media and Volunteers-NGO categories. Individuals were the category most cited by all other categories except for Institution; this maybe shows a certain reticence by a public agency to endorse messages from sources they cannot verify. Institutions tend mainly to cite (mention and retweet) other institutions, in other words sources that are well recognized as trustworthy. The key results for each channel are presented.

In #LIG channel Institution resulted as the category that cited less, a sign of a classic “broadcast” use of social media like Twitter, mainly managed as a medium for publishing information and not as a means to interact with the public. Media were mainly cited by media accounts themselves; this tendency to cite accounts belonging to the same class of users also emerges among other categories, where category acts as a sub-community where Twitter authors refer to each other. Media and Individuals mentioned Weather accounts that in turn were not so active, they mainly mentioned Individuals and other weather sources.

In #PIE channel individuals were the most active in mentioning and retweeting. Despite what happens in #LIG, Individuals mentioned mostly Weather accounts only secondly other citizens, media, and very few institutions and NGOs. Weather also turned out to be the most cited class by all the others, also due to a very poor presence of institutions, not tweeting on codified hashtags. This inactivity of institutions on the #PIE channel may be explained by the fact that during the severe weather events in Piedmont in October/November 2014 there were two conflicting codified hashtags #allertameteoPIE and #allertameteoPM (the dataset counts 2192 occurrences of #allertameteoPM and 846 of #allertameteoPIE), many institutions also using the latter. Institutions made no citation of other institutions. Adoption of the codified hashtag in Piedmont was for this reason very weak.

In #TOS channel Individuals, very active in mentioning and retweeting, cited mostly institutions. #TOS was the only channel where institutions had this active presence within the codified hashtags community. Weather class was the most retweeted by Individuals while Institution was the most mentioned, in a call for action by the public. Institution was the most cited class of users, followed by Weather. It is also interesting to note that institutions cited firstly other institutions and secondly weather’s. The #allertameteoTOS promoted by the regional weather service (@flash_meteo) and sustained by municipalities and local civil protection accounts was in this way codified as the official hashtag to be used for weather warning in Tuscany region.

**3.4 Content analysis**

Content analysis was meant to understand if codified hashtags were an effective communication channel to aggregate formal and informal information during emergencies. On this purpose, authors coded native tweets in different content categories relevant for situational awareness and analysed their distribution and activity of different classes of users in the three contexts. Only a Refined DataSet (as explained in section 2.3) was considered for content analysis. Our analysis showed that codified hashtags were able to aggregate tweets focused on situational updates: messages categories contributing to situational awareness represents around the 85% of the RDS, with minor differences in the three context (84% in #PIE and #LIG and 95% in #TOS). This is almost twice the size of results of Vieweg study^12^ on Red River Floods, reporting 49% of on-topic tweets being situational updates. Even in the case of Liguria floods, the messages conveyed through #allertameteoLIG were focused on situational updates. Individuals/citizens have proven so careful to use the correct hashtag, and many have posted tweets with exhortations to use it strictly for messages concerning emergency management. The most frequently occurring categories of tweets in the codified hashtags RDS were messages of Hazard Location, Weather, Resources, Transport Conditions, Warnings and Advice, as showed in Table 5.

**Table 5: Distribution of content in the three channels:** Distribution of contents by categories in the three channels.

	Total	#LIG	#PIE	#TOS
Advice	7%	8%	5%	5%
Comments	12%	14%	4%	2%
Damage/Injury	5%	5%	3%	3%
Evacuation/Closures	6%	5%	4%	11%
Hazard Location	24%	24%	25%	20%
News Report	3%	2%	11%	3%
Reassurance	2%	2%	3%	2%
Resources	15%	17%	12%	10%
Transport Conditions	9%	9%	8%	9%
Warning	6%	5%	3%	13%
Weather	12%	10%	22%	22%

An analysis of RDS illustrates its main features and shows similarities and divergences in the three contexts. Around 24% of tweets were coded as *Hazard Location*. Abundance of Hazard Location messages (situational updates of the event containing a geo location reference) confirms that social media have a role in information exchange and that during a disaster individuals provide important updates to complement official information. Hazard location was also the category with the greatest number of retweets.

Messages coded as *Weather* accounts for 12%, but in #LIG they are half the size (10%) of #TOS and #PIE (22%). A reason could be that in Liguria the regional weather service (ARPAL) was not using the codified hashtag in tweets; Twitter account @ARPAL_meteomare contributed posting automatic updates when a new weather monitoring was issued (on the institutional web site www.arpal.gov.it). Citizens reposted those references by adding the codified hashtags, acting as a hub between trusted sources and the hashtag community. Those kind of tweets were coded as Resources, because the message did not contain any clear textual update but only an external reference. In fact in #LIG dataset Resources category is higher than in #TOS and #PIE. A lot of Individuals published weather related messages but it’s worth to notice that writing a textual update within the tweet is by far more useful for people following the Twitter stream than a mere URL sharing that requires the user to open another application to get the information.

About 9% of tweets were coded as *Transport Conditions* (with similar reach in the three channels), a percentage that is three times the size of what reached by the same category of tweets in Red River Floods case study 12 . In Tuscany this tweets were mostly published by Institutions (78% of Transport Conditions tweets), while in #LIG and #PIE they were mostly coming from individuals (88% and 48%). In Tuscany the existence of an official Twitter account (@muoversintoscana) dedicated to information on the regional road network made it possible to have a continuous and reliable flow of information, also labelled by the hashtag #viabiliTOS.

A 6% of tweets was coded as *Warning* in the RDS but with interesting differences: 13% of the #TOS channel stream and only 3% of #PIE and 5% of #LIG. A percentage that is in line with previous studies, like the Red River floods by Vieweg (2010)^12^ , and much higher of the tiny 0,31% reached by warning tweets collected during Yolanda Typhoon in Soriano (2016)^47^ . These were the tweets announcing the issuing of a weather warning; the high percentage of tweets in Tuscany it’s not a result of higher warnings but rather an outcome of sharing every weather alert on Twitter by the #allertameteoTOS hashtag. Main contribution was by @flash_meteo, the regional weather service, and other Weather accounts: 20% of tweets published by Weather users in #TOS were Warnings, three times as many compared to what happened in #LIG and #PIE (6% and 3% of tweets published by users belonging to Weather related accounts).

Tweets coded as *Reassurance* were 2% of the dataset, with no quantitative disparity in the three contexts but with different classes of users providing the message. Reassurance is an important class of messages aimed at informing the public that first responders are prepared and active during the event. One would expect a primary role of the institutions called upon to reassure the public that the emergency is under control, but in #LIG dataset these tweets were mainly coming from Volunteers and Individuals (67% and 16% of #LIG Reassurance tweets) rather than Institutions (8%). On the other hand, in #TOS were primarily Institutions to publish Reassurance kind of messages: 35% of tweets coded as Reassurance had institutions as authors, followed by Media users (29%) and individuals accounts (24%).

*Evacuation/Closures* messages (updates on evacuation procedures and closures of schools and public offices) accounts for 6% of RDS. They represent important information for the public and are usually issued by institution and republished by local and national media. In #LIG authors of those messages were mainly individuals (92% of Evacuation tweets), in #PIE Media (45%) and individuals (38%) while in #TOS authors of Evacuation messages have been mainly Media (51%) and institutional (29%) accounts.

A small percentage of tweets were coded as *Damages*, around 5%. While in Liguria these tweets were around 300 in #TOS and #PIE were barely 25. Individuals were the main authors of tweets coded as Damages in #LIG, confirming the important role of the public as information provider on the ground.

*Advice* messages amount at 7% of the RDS. These are tweets informing on how to cope with the emergency, giving safety precautions, but also including local numbers to call during emergency and advices on how to properly tweet and use the hashtags. In #LIG 86% of these tweets had Individuals as authors and just 3% Institutions; in #TOS 25% of authors of Advice tweets were Institutional account. In #PIE Institution made no Advice tweets at all.

Tweets not contributing to situational awareness, but still on-topic, were coded in two categories, *News Report*, that correspond to tweets about news and media coverage of the emergency; and the category *Comments*, messages expressing personal opinion, emotions or blames. In #TOS and #PIE this category was scarce (4% and 2% of tweets), while in #LIG Comments were the third most numerous category with 14% of tweets. Because of the damages caused by Genoa Flood, in Liguria people used Twitter to complain over local government and politicians. As well in Tuscany Twitter functioned as a digital agorà where citizens protested over the mayor and the municipality of the city of Carrara, but critics were not conveyed through the codified hashtag stream, other hashtags were used like #alluvioneCarrara (Carrara flood) and #carrarasiribella (Carrara rebels), the last one being a specific tag to organize the public protest. This confirms a more aware use of #allertameteoTOS by the Tuscan community in general.

Compared to similar studies on Twitter usage during flood emergency 11 ^,^ 12 , in the RDS dataset tweets including geo-location information are a higher percentage of all on topic tweets; they are no less than 38% of all tweets (considering that messages coded as Hazard Location, Transport Conditions and Evacuation have always a location reference), that is much more of the 18% reported for Red River study 12 . Tweets that include information about the location of people, the local impact of the hazard, or evacuation sites can improve a better understanding of the situation for individuals reading those messages. Geo-location information is also very useful for the automatic retrieval of relevant information during disasters.

**3.5 Hashtags**

A last metrics that was analysed concerning hashtags. The channels considered in this work are generated by the codified hashtags for weather warning, so in this dataset each tweet by default contains at least one hashtag. Table 6 presents the distribution of tweets by number of hashtags included in each tweet. It is self-evident that very few choose to limit hashtagging to the codified one; the majority of users added on average one or two more hashtags to the tweets; around 30% (23% in LIG; 39% in TOS and 32% in PIE) included 3 or more hashtags in the tweet, up to 12 in one tweet.

**Table 6: number of tweets by number of hashtags included in the tweet:** 

Channel	0#	1#	2#	3#	4# or more
#LIG	63	2606	1884	856	506
#TOS	6	241	316	205	148
#PIE	2	219	254	102	126

The reasons for this behaviour could be different and we may suppose that codified hashtags use was at a very early stage in November 2014 (it still is in some italian regions), and people choose to combine it with other hashtags emerging from the ground (like #alluvionegenova or #alluvioneCarrara, included in the top hashtags of the channel); many users also used more than one codified hashtag in the same tweet to reach both communities, especially for messages about the weather forecast. We could also argue that even in the presence of a keyword used to tag a specific conversation and community, as in this case, people tend to introduce more hashtags to highlight information they consider critical inside the tweet, like for example a place or a street or even a person. To answer to this question and better understand hashtagging practices we coded hashtags to gain more knowledge on communication habits. Hashtags included in the channel were annotated in ten categories following the criteria explained in Methodology; categories were then ranked on the basis of frequency.

**Distribution of hashtags following the annotated categories (native tweets only):**
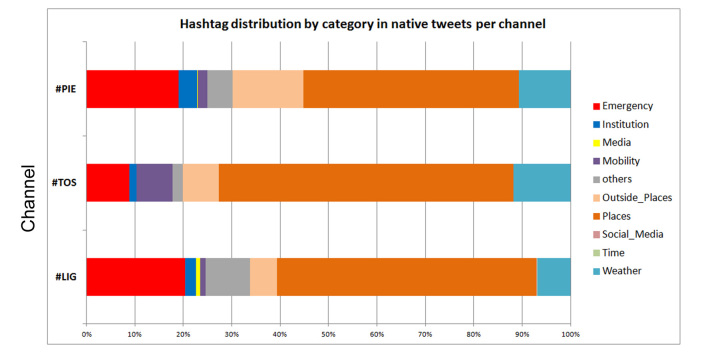

As shown in Figure 6 in all three channels the majority of hashtags, around 50%, belong to the class Places. This is in line with use of Twitter during emergencies to share information about what is happening on the ground, point out problems or report damage, giving specific geographic indications. Tweets containing pictures also tend to include geographic information. The use of geographic information within the codified hashtags community is very important given that a very small percentage of tweets are geo-located and the technique of obtaining location information by querying the users profile is very imprecise. A sub-class Outside_places recorded hashtags referring to a location external to the regional geographic domain of the codified hashtags; references outside the region are about 10-30% of the class Places. It can be said that tweets were very focused on the local context.

The second most numerous class of hashtags used is Emergency, higher in #LIG and #PIE channel (around 20% of total hashtags used) half that in #TOS channel; this is probably due to the destructive impact of bad weather in Liguria, with the flash floods in Genoa and Chiavari. Another element to be considered is the higher number of users involved in #LIG channel, almost 6000 compared to less than a thousand in the other two channels; different users means different language, different ways to describe an emergency situation. Weather was of course another very popular class of hashtags; weather accounts were very active in all the channels and severe weather was by default the main topic of the codified hashtags community. So it is no surprise that many hashtags belong to the class Weather. The hashtag category related to Mobility is also quite numerous; indeed during emergencies users tend to share information about the state of the roads. In Tuscany one of the top authors was the account @muoversintoscan (Tuscanmobility), which shared a lot information about traffic and transport problems. Media was a well- represented class of hashtags in #LIG but very little used in the other two channels, due to the involvement of PrimoCanale, a local TV station based in Genoa that also fully covered the emergency on Twitter by means of its account @primocanale and the account of its main news presenter. Hashtags referred to Institutions were quite represented in #LIG channel, not in #TOS and #PIE, maybe due to the fact that Twitter institutions accounts were not active in the codified hashtag community nor in Twitter, as a result many users (mainly citizens) quoted them in their tweet also in terms of blame. For the Genoa flash flood there were many critics of the Genoa mayor and Liguria Governor in the media and also on Twitter, and this also emerges in hashtagging practice. Very few hashtags were related to Time category, this kind of information seems not to be considered as key to become a hashtag.

## Conclusions

This paper presents some findings about effective use of codified hashtags on Twitter during emergencies by analyzing a dataset of tweets based on three regional codified hashtags for weather warning in Italy. It is an exploratory work to assess if codified hashtags may contribute in aligning formal and informal sources of information during emergencies. In particular we wanted to investigate: the propagation of codified hashtags in the three different regions (Liguria, Tuscany and Piedmont); the responsive use of the hashtag by classes of users playing a role during emergencies; the hashtagging practices emerging; the categories of information exchanged on Twitter by mean of those hashtags.

Due to modest Twitter diffusion in Italy the three datasets collected are quite small compared to similar datasets examined in previous works about social media use in natural disasters as in the United States or Australia (#hurricaneSandy, #qldfloods). Although small, the main citation patterns of this dataset, like retweeting behavior and number of tweets with URLs, are in line with those reported in the literature for crisis events 39 .

The coding of users, hashtags and messages made it possible to make evaluations on the flow of information exchanged within the hashtag community and highlight the role of different users, particularly of institutional accounts which represent main trusted sources. Codified hashtag have proven to be an effective channel of communication with a great majority of information exchanged being related to situational updates, like hazard location, flood updates, damages or injuries reports, road and transports conditions, weather updates, evacuation and closures messages. All this situational updates communicated on Twitter may contribute to situational awareness during an emergency.

Citizens confirmed to be the most active class of users, a peculiarity of crisis events also expressed in the extraordinary retweeting behavior, especially in Liguria theatre of several flash floods during the period considered. Tuscany was the region were Institutions, mainly local civil protection offices, played a central role. In the #allertameteoTOS channel 22% of native tweets were published by Institutions, ten times more than in #allertameteoLIG (only 1%) and in #allertameteoPIE (2%). This active role of institutional made Tuscany the region where the weather warning codified hashtag gained widest official agreement, fundamental for a wider and concrete adoption in an emergency. Piedmont was the region where the use of codified hashtag was weakest with a lot of tweets posting information about Liguria (the third most used hashtag in the #PIE dataset was #allertameteoLIG). The two versions of the regional codified hashtag (#allertameteoPIE and #allertameteoPM) were a symptom of this less structured approach.

Hashtags coding revealed an extraordinary percentage of hashtags related to geographic names (50% of items belongs to category Places) also confirmed in the content analysis of tweets. Emergency was the second most represented category of hashtags. In #TOS it was a smaller category than in the other two regions, possibly a sign that in Tuscany the semantic related to “Emergency” was felt to have been accomplished by the codified hashtag. Hashtagging practice anyhow reveals a high degree of fragmentation, even higher in the case of wide participation of members of the public (like in #LIG channel), thus making the practice of adopting and promoting a codified hashtag even more reasonable and worthwhile to collect all information related to regional weather warning under a single umbrella.

Content analysis of tweets showed an almost null rate of off-topic tweets and a very high rate (91%) of tweets related to situational updates, demonstrating that codified hashtags could be very effective in conveying relevant information during disasters, aligning formal and informal sources of information. Compared to similar works, #allertameteoXXX dataset count a very high percentage of information related to situational updates, 84% of native tweets, compared for example to 56% reported for Thai floods case study in Konghton 2012^11^ , or 61% for Red River floods in Vieweg 2010^12^ .

Finally when considering the possibility to use computer systems to automatically extract information from micro-blogging messages during disasters, as investigated by many researchers, the use of codified hashtags could potentially improve considerably the quality of information extraction due to high rate of on-topic messages and local updates. On this purpose, institutions and emergency managers shall consider carefully to support the adoption of codified hashtags in preparedness activity as a good practice to inform citizens on how to tweet and use hashtags in an effective way. In Tuscany were many institutions adopted effectively the #allertameteoTOS, the percentage of tweets focused on situational updates was up to 95%.

Codified hashtags will function if institutions support them before, during and after the emergency; thus they may contribute to filter relevant information from the immense Twitter sphere. This will also potentially reduce the amount of information lost because citizens, authorities and the media had not reached an agreement on how to label conversations.

## Appendix 1

A table with the three most retweeted messages of the three channels is provided.

**Table 1. Most retweeted messages in the three channels:** 

Channel	Messages	Retweets count	Author	Users Category	Date
#LIG	pazzesco il #polcevera a distanza di pochi minuti foto di @stefaniaconti #allertameteolig #alluvionegenova http://t.co/gesfgwlzsp	362	@farmaciaserrage	Individuals	2014-11-15
#LIG	polcevera, fiumara, sampierdarena. parole non ce ne sono più. #allertameteolig #alluvionegenova #genova @emergenza24 http://t.co/7ru4on5z0n	177	@micky_genova	Individuals	2014-11-15
#LIG	#allertameteolig testimone #voltri "boato dal #cerusa, distrutte fabbriche sugli argini. non uscite" @ilsecoloxix http://t.…	145	@gtimossi	Individuals	2014-11-15
					
#TOS	alluvione a #chiavari. massima allerta anche per la toscana ►http://t.co/0nixdbq5ik #allertameteolig #allertameteotos http://t.co/dgvkmg9tgc	94	@3BMeteo	Weather	2014-11-10
#TOS	#allertameteotos: domani allerta arancio per piogge e temporali su tutta la toscana, rossa su bacino albegna (gr) http://t.co/qvwlgzwup4	29	@flash_meteo	Weather	2014-11-04
#TOS	#allertameteolig #allertameteotos temporali in corso sul levante e in mare di fronte a la spezia e litorale toscano http://t.co/1fwk7okrwi	28	@AllertaMeteoLIG	Weather	2014-11-09
					
#PIE	ultim'ora http://t.co/wm9qgrkbse allerta 2 fino a stasera tra liguria e piemonte #allertameteopie #allertameteolig http://t.co/njtuhaqtjm	45	@3BMeteo	Weather	2014-11-15
#PIE	mappa piene fluviali. previsioni del 30 novembre #allertameteopie #allertameteopm http://t.co/ajy5bxra98	23	@ArpaPiemonte	Weather	2014-11-30
#PIE	esaurimento delle precipitazioni nella notte. livelli idrometrici ancora in crescita http://t.co/a6go0ern9g #allertameteopm #allertameteopie	21	@ArpaPiemonte	Weather	2014-11-15

## Competing Interests

The authors have declared that no competing interests exist.

## References

[1] Fraustino, J. D., Brooke, L., & Yan, J. (2012). Social Media Use during Disasters: A Review of the Knowledge Base and Gaps, Final Report to Human Factors/Behavioral Sciences Division. Science and Technology Directorate, US Department of Homeland Security. College Park, MD

[2] Seeger, M. W. (2006). Best practices in crisis communication: An expert panel process. Journal of Applied Communication Research, 34(3), 232-244

[3] Veil, S. R., Buehner, T., & Palenchar, M. J. (2011). A Work‐In‐Process Literature Review: Incorporating Social Media in Risk and Crisis Communication. Journal of contingencies and crisis management, 19(2), 110-122.

[4] Sutton, J., Palen, L., & Shklovski, I. (2008, May). Backchannels on the front lines: Emergent uses of social media in the 2007 southern California wildfires. In Proceedings of the 5th International ISCRAM Conference (pp. 624-632). Washington, DC

[5] Yates, D., & Paquette, S. (2011). Emergency knowledge management and social media technologies: A case study of the 2010 Haitian earthquake. International Journal of Information Management, 31(1), 6-13.

[6] Smith, B. G. (2010). Socially distributing public relations: Twitter, Haiti, and interactivity in social media. Public Relations Review, 36(4), 329-335.

[7] Bruns, A., & Burgess, J. (2014). Crisis Communication in Natural Disasters: The Queensland Floods and Christchurch Earthquakes. In P. Weller, Katrin, Bruns, Axel, Burgess, Jean, Mahrt, Merja (Ed.), Twitter and Society (pp. 373–384). New York: Cornelius (EDS) Peter Lang. Retrieved from

[8] Hughes, A. L., St Denis, L. A., Palen, L., & Anderson, K. M. (2014) Online public communications by police & fire services during the 2012 Hurricane Sandy. In Proceedings of the SIGCHI Conference on Human Factors in Computing Systems (pp. 1505-1514). ACM

[9] Hughes, A. L., St Denis, L. A., Palen, L., & Anderson, K. M. (2014) Online public communications by police & fire services during the 2012 Hurricane Sandy. In Proceedings of the SIGCHI Conference on Human Factors in Computing Systems (pp. 1505-1514). ACM.

[10] Dashti, S., Palen, L., Heris, M. P., Anderson, K. M., Anderson, S., & Anderson, S. (2014). Supporting disaster reconnaissance with social media data: a design-oriented case study of the 2013 colorado floods. Proc. of ISCRAM.

[11] Kongthon, A., Haruechaiyasak, C., Pailai, J., & Kongyoung, S. (2012, July). The role of Twitter during a natural disaster: Case study of 2011 Thai Flood. InTechnology Management for Emerging Technologies (PICMET), 2012 Proceedings of PICMET'12: (pp. 2227-2232). IEEE.

[12] Vieweg, S., Hughes, A. L., Starbird, K., & Palen, L. (2010, April). Microblogging during two natural hazards events: what twitter may contribute to situational awareness. In Proceedings of the SIGCHI conference on human factors in computing systems (pp. 1079-1088). ACM.

[13] Colley, K. L., & Collier, A. (2009). An overlooked social media tool? Making a case for wikis. Public Relations Strategist, 34-35.

[14] Hagar, C., & Haythornthwaite, C. (2005). Crisis, farming & community. The Journal of Community Informatics, 1(3).

[15] Palen, L., Vieweg, S., Liu, S. & Hughes, A. (2009). Crisis in a Networked World: Features of Computer- Mediated Communication in the April 16, 2007 Virginia Tech Event. Social Science Computing Review, Sage, 27 (4), pp. 467-480.

[16] Palen, L., Anderson, K. M., Mark, G., Martin, J., Sicker, D., Palmer, M., & Grunwald, D. (2010, April). A vision for technology-mediated support for public participation & assistance in mass emergencies & disasters. In Proceedings of the 2010 ACM-BCS visions of computer science conference (p. 8). British Computer Society.

[17] Chang, H. C. (2010). A new perspective on Twitter hashtag use: diffusion of innovation theory. Proceedings of the American Society for Information Science and Technology, 47(1), 1-4.

[18] Wukich, C. and Steinberg, A. (2013), Nonprofit and Public Sector Participation in Self-Organizing Information Networks: Twitter Hashtag and Trending Topic Use During Disasters. Risk, Hazards & Crisis in Public Policy, 4: 83–109. doi: 10.1002/rhc3.12036.

[19] Suh, B., Hong, L., Pirolli, P., & Chi, E. H. (2010, August). Want to be retweeted? large scale analytics on factors impacting retweet in twitter network. In Social computing (socialcom), 2010 ieee second international conference on (pp. 177-184). IEEE.

[20] Dwarakanath, L. (2014). # Emergency : Role of Twitter Hashtags during and after a disaster, 3(4), 948–953.

[21] Meier P., (2014), The Filipino Government’s Official Strategy on Crisis Hashtags, http://irevolution.net/2014/07/01/filipino-official-strategy-crisis-hashtags/ accessed on 27/02/2015

[22] UNOCHA (2014), Hashtag Standards For Emergencies, United Nations Office for the Coordination of Humanitarian Affairs, OCHA Policy and Studies series, October 2014 | 012

[23] Blog post “20 hashtags per una protezione civile partecipata” http://capitanachab.tumblr.com/ accessed on 20th November 2015

[24] Parisi L., Comunello F., Amico A. (2014). #allertameteoSAR: analisi di un hashtag di servizio tra dinamiche di influenza e nuove forme di engagement. In Francesca Comunello (a cura di), Social media e comunicazione d’emergenza, Guerini, Milano 2014.

[25] Giglietto, F., & Lovari, A. (2013). Amministrazioni pubbliche e gestione degli eventi critici attraverso i social media: il caso di# firenzeneve. Mediascapes journal, (1), 99-118.

[26] Olteanu, A., Vieweg, S., & Castillo, C. (2015). What to Expect When the Unexpected Happens: Social Media Communications Across Crises. In In Proc. of 18th ACM Computer Supported Cooperative Work and Social Computing (CSCW’15), (No. EPFL-CONF-203562).

[27] ERCC web portal: http://erccportal.jrc.ec.europa.eu/

[28] Aeronautica Militare Italiana, (2014), Bollettino Mensile Novembre 2014, web site accessed on 3rd December 2015

[29] ARPA, Agenzia Regionale Protezione Ambiente , (2014), Rapporto tecnico evento 3-6 novembre 2014, website accessed on 3rd December 2015

[30] ARPA - Agenzia Regionale Protezione Ambiente, (2014), Rapporto tecnico evento 9-17 novembre 2014, website accessed on 3rd December 2015

[31] ARPAL CENTRO FUNZIONALE METEO-IDROLOGICO DI PROTEZIONE CIVILE DELLA REGIONE LIGURIA,(2014), Rapporto Evento Alluvione genova, web site accessed on 3rd December 2015

[32] Consorzio LaMMA Regione Toscana CNR, (2014), Report Evento 10-11 novembre 2014, website accessed on 3rd December 2015

[33] Consorzio LaMMA Regione Toscana CNR, (2014), Report Evento 4-5 novembre 2014, website accessed on 3rd December 2015

[34] Silvestro, F., Rebora, N., Giannoni, F., Cavallo, A., Ferraris, L., The flash flood of the Bisagno Creek on 9th October 2014: an “unfortunate” combination of spatial and temporal scales. Journal of Hydrology, 2015 doi:10.1016/j.jhydrol.2015.08.004.

[35] Github pages: https://github.com/alfcrisci/rTwChannel

[36] Lin, Y. R., Margolin, D., Keegan, B., Baronchelli, A., & Lazer, D. (2013). # Bigbirds Never Die: Understanding Social Dynamics of Emergent Hashtag. arXiv preprint arXiv:1303.7144.

[37] Yang, L., Sun, T., Zhang, M., & Mei, Q. (2012, April). We know what@ you# tag: does the dual role affect hashtag adoption?. In Proceedings of the 21st international conference on World Wide Web (pp. 261-270). ACM.

[38] Zappavigna, M. (2015). Searchable talk: the linguistic functions of hashtags. Social Semiotics, 25(3), 274-291.

[39] Bruns, A., Highfield, T., & Lind, R. A. (2012). Blogs, Twitter, and breaking news: The produsage of citizen journalism. Produsing Theory in a Digital World: The Intersection of Audiences and Production in Contemporary Theory, 80(2012), 15-32.

[40] Palen, L., & Liu, S. B. (2007). Citizen communications in crisis: anticipating a future of ICT-supported public participation. In Proceedings of the SIGCHI conference on Human factors in computing systems (pp. 727-736). ACM.

[41] Bruns, A., & Stieglitz, S. (2014). Metrics for Understanding Communication on Twitter. In Twitter and Society (pp. 69–82). Retrieved from http://www.peterlang.com/index.cfm?event=cmp.ccc.seitenstruktur.detailseiten&seitentyp=produkt&pk=71177&cid=5&concordeid=312169 accessed on 3rd December 2015

[42] Bonnan-White J, Shulman J, Bielecke A. Snow Tweets: Emergency Information Dissemination in a US County During 2014 Winter Storms. PLOS Currents Disasters. 2014 Dec 22 . Edition 1. doi: 10.1371/currents.dis.100a212f4973b612e2c896e4cdc91a36. 10.1371/currents.dis.100a212f4973b612e2c896e4cdc91a36 PMC4323415 25685629

[43] Starbird, K., Palen, L., Hughes, A. L., & Vieweg, S. (2010). Chatter on the red: what hazards threat reveals about the social life of microblogged information. CSCW ’10 Proceedings of the 2010 ACM Conference on Computer Supported Cooperative Work, 241–250. doi:10.1145/1718918.1718965

[44] Crisis Commons web site: https://crisiscommons.org

[45] Starbird, K., & Palen, L. (2010). Pass it on?: Retweeting in mass emergency. Proceedings of the 7th International ISCRAM Conference, (December 2004), 1–10. doi:10.1111/j.1556-4029.2009.01231.x

[46] Sutton, J., Spiro, E. S., Johnson, B., Fitzhugh, S., Gibson, B., & Butts, C. T. (2014). Warning tweets: serial transmission of messages during the warning phase of a disaster event. Information, Communication & Society, 17(6), 765–787. doi:10.1080/1369118X.2013.862561

[47] Soriano, C. R., Roldan, M. D. G., Cheng, C., & Oco, N. (2016). Social media and civic engagement during calamities: the case of Twitter use during typhoon Yolanda. Philippine Political Science Journal, 4451(February), 1–20. doi:10.1080/01154451.2016.1146486

[48] Cohen, J. (1960). "A coefficient of agreement for nominal scales". Educational and Psychological Measurement 20 (1): 37–46. doi:10.1177/001316446002000104

